# Acute Gastric Dilatation With Ischemia and Perforation Requiring Emergency Total Gastrectomy: A Case of Suspected Abdominal Compartment Syndrome

**DOI:** 10.7759/cureus.41265

**Published:** 2023-07-01

**Authors:** Shariful Islam, Aneela Shah, Vijay Naraynsingh, Patrick Harnarayan

**Affiliations:** 1 Laparoscopic and Oncoplastic Breast Surgery, San Fernando General Hospital, San Fernando, TTO; 2 Department of Clinical Surgical Sciences, University of the West Indies, St. Augustine, TTO; 3 General Surgery, San Fernando General Hospital, San Fernando, TTO; 4 Surgery, Medical Associates Hospital, St. Joseph, TTO; 5 Department of Clinical Surgical Sciences, University of The West Indies, St. Augustine, TTO

**Keywords:** gastric perforation, gastric ischemia, abdominal compartment syndrome, gastric volvulus, acute gastric dilatation

## Abstract

Acute gastric dilatation is an uncommon surgical pathology, leading to gastric ischemia, necrosis, perforation, sepsis, and death if untreated. While rare, the development of abdominal compartment syndrome is also a devastating complication of this entity. We present a case of a 42-year-old male with a history of gastric volvulus, presenting with severe acute abdominal distension and multi-organ failure. A diagnosis of acute gastric dilatation was made, with suspicion of abdominal compartment syndrome. Emergency laparotomy was performed when nasogastric decompression failed. Total gastrectomy without anastomosis was performed due to the patient’s hemodynamic instability. However, he demised shortly after on the operating table. This case report demonstrates that even with rapid diagnosis and management, acute gastric dilatation continues to be associated with high mortality.

## Introduction

First described in 1883, acute gastric dilatation is an uncommon disorder, with most documentation of its occurrence in the surgical literature being case reports [1]. It is associated with a variety of causes, and despite a rich collateral blood supply, massive gastric dilatation can predispose to ischemia, necrosis, and perforation. Additionally, severe gastric distension predisposes patients to sustained intra-abdominal hypertension and abdominal compartment syndrome when multiorgan dysfunction subsequently occurs.

While patients typically present with vague abdominal pain, distension, and nausea, confirmation of the diagnosis requires a high degree of clinical suspicion in addition to radiological imaging identifying a severely distended stomach. Initial management is centered on gastric decompression via a nasogastric route, as well as intravenous fluid resuscitation and multiorgan support. However, laparotomy becomes necessary when tube decompression is unsuccessful or when gastric necrosis or perforation occurs.

We present a case of acute gastric dilatation in a male patient with a history of organoaxial gastric volvulus and clinical signs suggestive of abdominal compartment syndrome. Initial tube decompression was unsuccessful and emergent laparotomy was undertaken for definitive management. This report places emphasis on the diagnosis and treatment options for this rare condition, highlighting the high mortality rate despite early intervention.

## Case presentation

A 42-year-old man was brought into the Accident and Emergency Department with severe abdominal pain and distension that began almost three hours prior. This was associated with acute shortness of breath and an inability to pass stool. He experienced no nausea, vomiting, or other gastrointestinal symptoms, but reported intermittent symptoms of abdominal distension over the previous three months.

The patient’s medical history included a diagnosis of Crohn’s Disease for which he was non-compliant with medical therapy. He reported being informed of having a “twisted stomach” at the age of twenty-four, identified during an upper gastrointestinal endoscopy. However, he refused treatment. He had no previous surgeries, was not on regular medication, and did not smoke or drink alcohol regularly. 

On examination, the patient appeared to be in respiratory distress and was cachectic with a severely distended abdomen. His vital signs included a blood pressure of 102/65mmHg, pulse of 140 beats per minute, respiratory rate of 35 breaths per minute, and oxygen saturation of 95% on room air.

**Figure 1:**
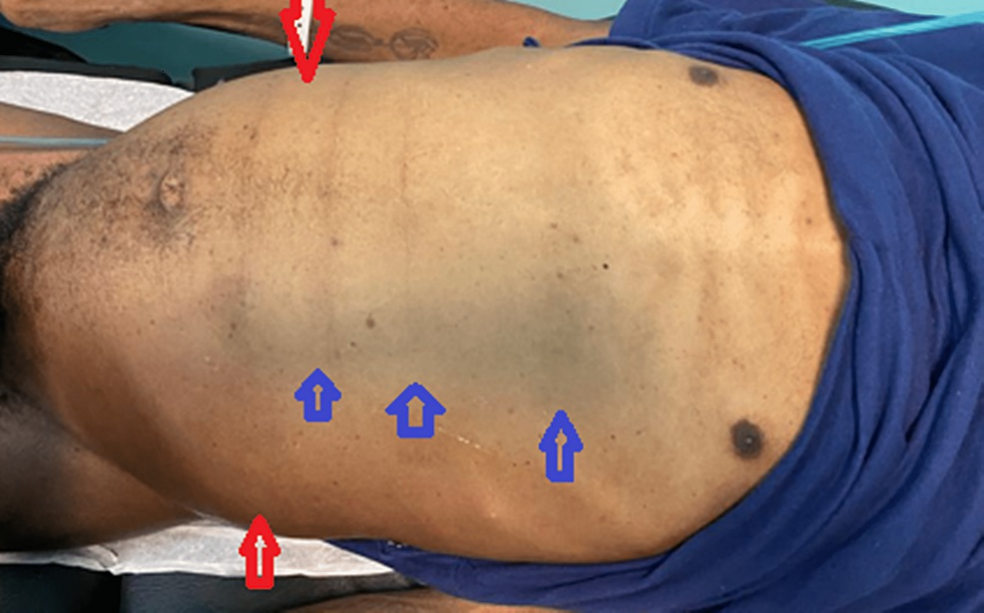
Picture of patient’s abdomen demonstrating severe abdominal distension (red arrows) and bluish discoloration of the skin (blue arrows)

A blue discoloration was noted on the left side of his abdomen, which was tense, distended, and displayed stone-like dullness to percussion (Figure 1).

Succussion splash was not appreciated, and bowel sounds were absent. Digital rectal examination and urinary catheter insertion were not possible due to high pressure causing resistance. Nasogastric tube advancement was also not possible due to resistance. Intravenous fluid resuscitation was started, with little improvement in his clinical status after two liters were infused.

A complete blood count showed hemoglobin of 12.3 g/dL, white cell count of 12 x 10^9^/L, and platelet count of 423 x 10^9^/L). However, he was hypokalemic (3.3 mEq/L) with an acute kidney injury (serum creatinine 2.2 mg/dL).

**Figure 2:**
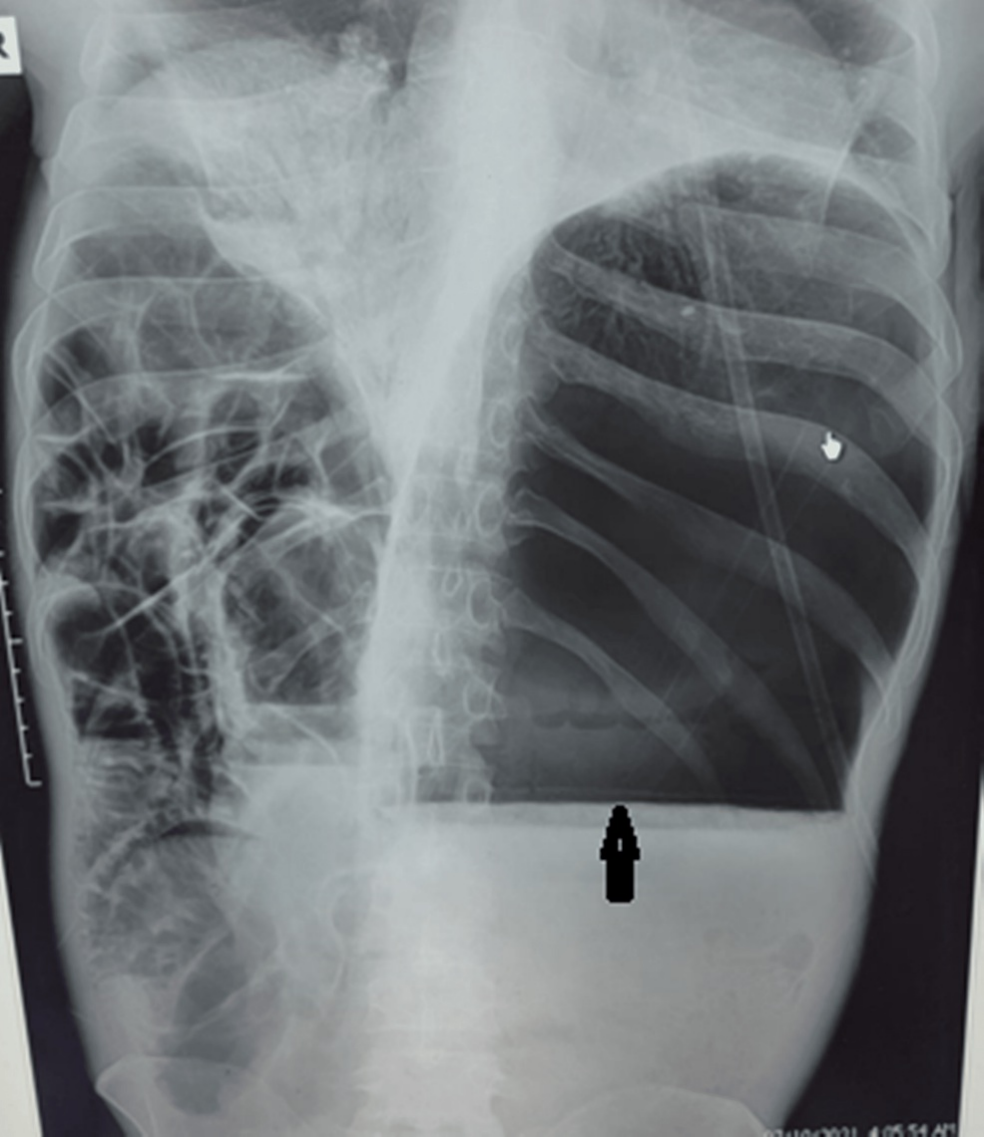
Erect X-ray of the abdomen showing severely distended stomach with fluid level (black arrow)

Arterial blood gas analysis revealed an uncompensated metabolic acidosis with a pH of 7.28, a base deficit of -12 mEq/L, and lactate of 5mmol/L. The patient was also hypoxemic, with PaO_2_ of 64mmHg. Erect abdominal X-rays demonstrated a significantly distended stomach with an air and fluid level (Figure 2). Computed Tomography (CT) was not pursued due to the patient’s hemodynamic instability.

A diagnosis of acute gastric dilatation was made, with suspicion of abdominal compartment syndrome, and the patient consented to an emergent laparotomy. At surgery, the patient was draped before anesthetic induction due to high suspicion of respiratory failure and difficult intubation. He was placed in a 30-degree head-up position for induction, and once asleep, a full midline incision was made to decompress the abdomen, facilitating intubation and ventilation. Inotropic support was initiated immediately afterward.

**Figure 3:**
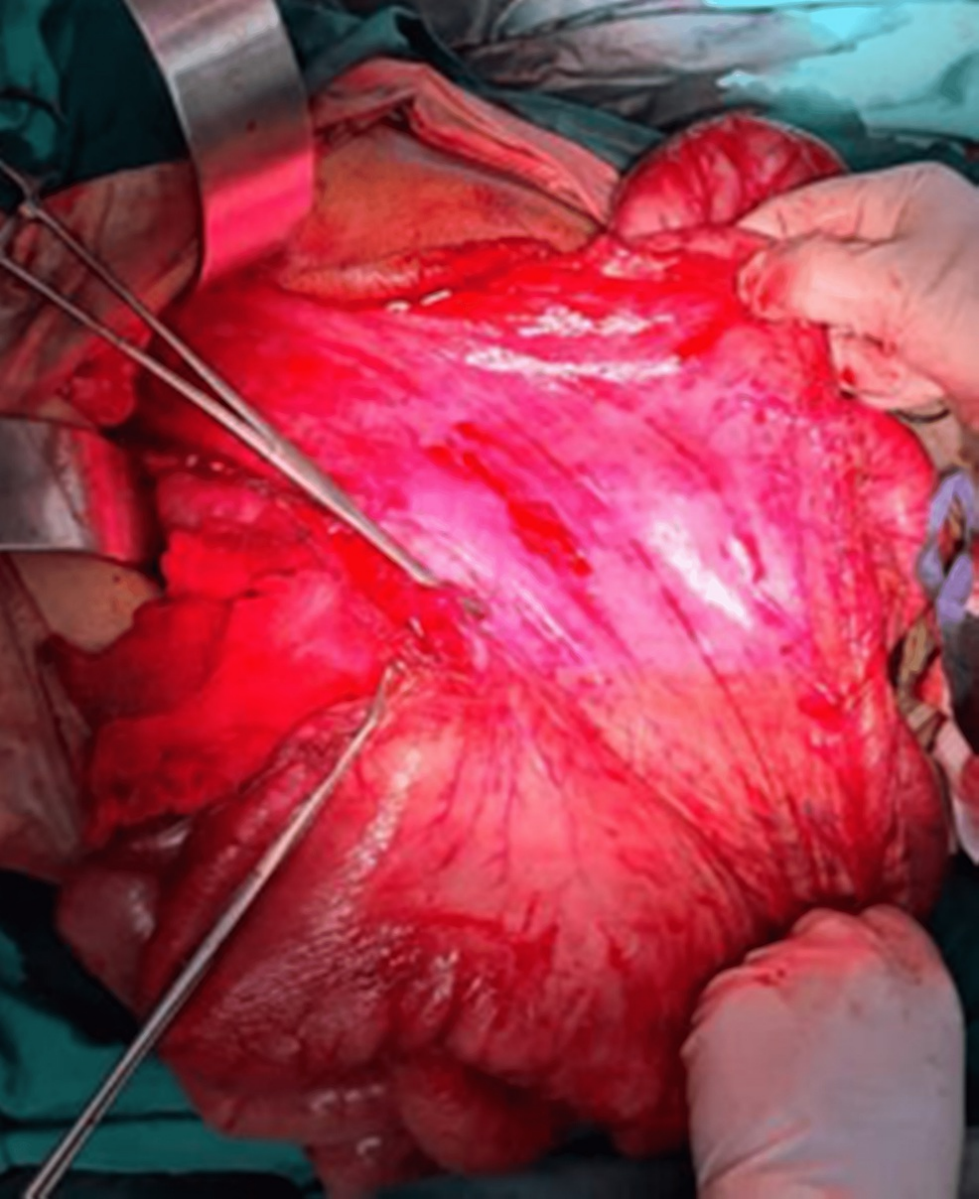
Intraoperative image showing massively dilated stomach (anterior view)

The small bowel rapidly eviscerated upon laparotomy and the ischemic, significantly distended stomach was identified as occupying the majority of the abdominal cavity (Figure 3).

**Figure 4:**
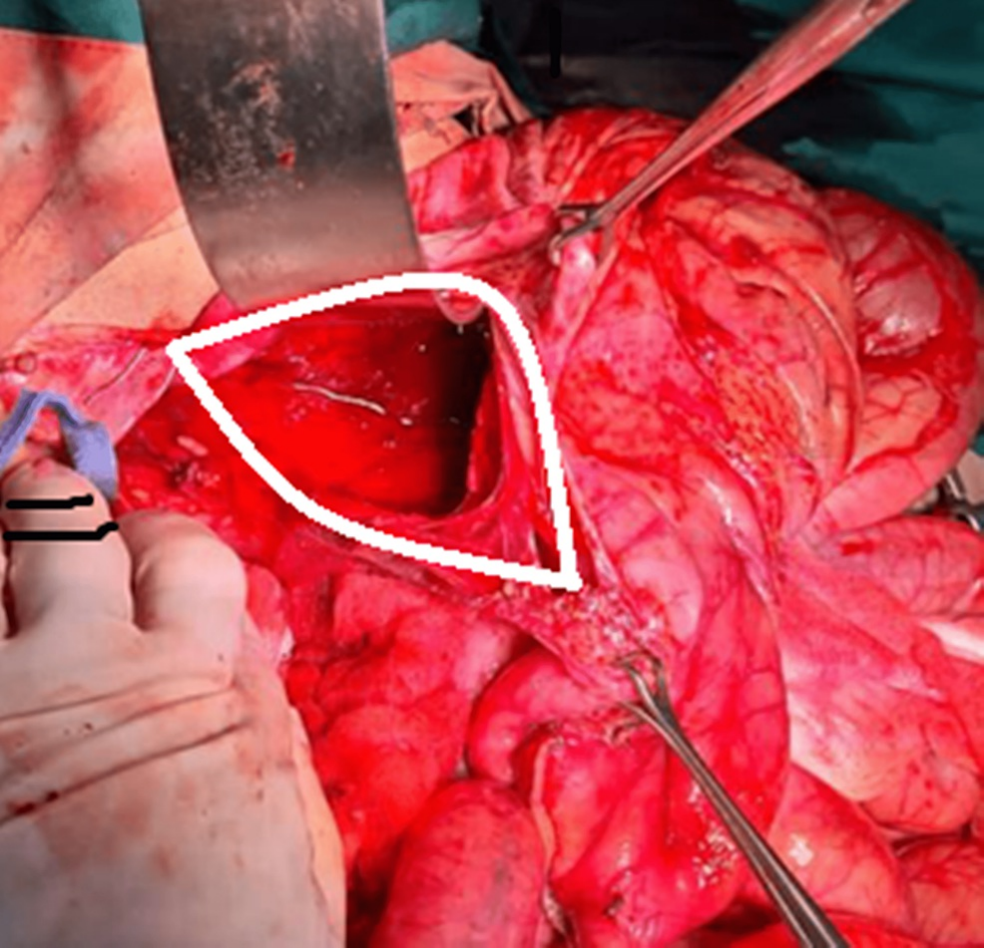
Intraoperative image showing large perforation at the lesser curvature of the stomach (the white elliptical area)

Immediately after opening the abdomen, the lesser curvature of the stomach ruptured spontaneously to release almost two liters of partially digested food material and old blood (Figure 4).

**Figure 5:**
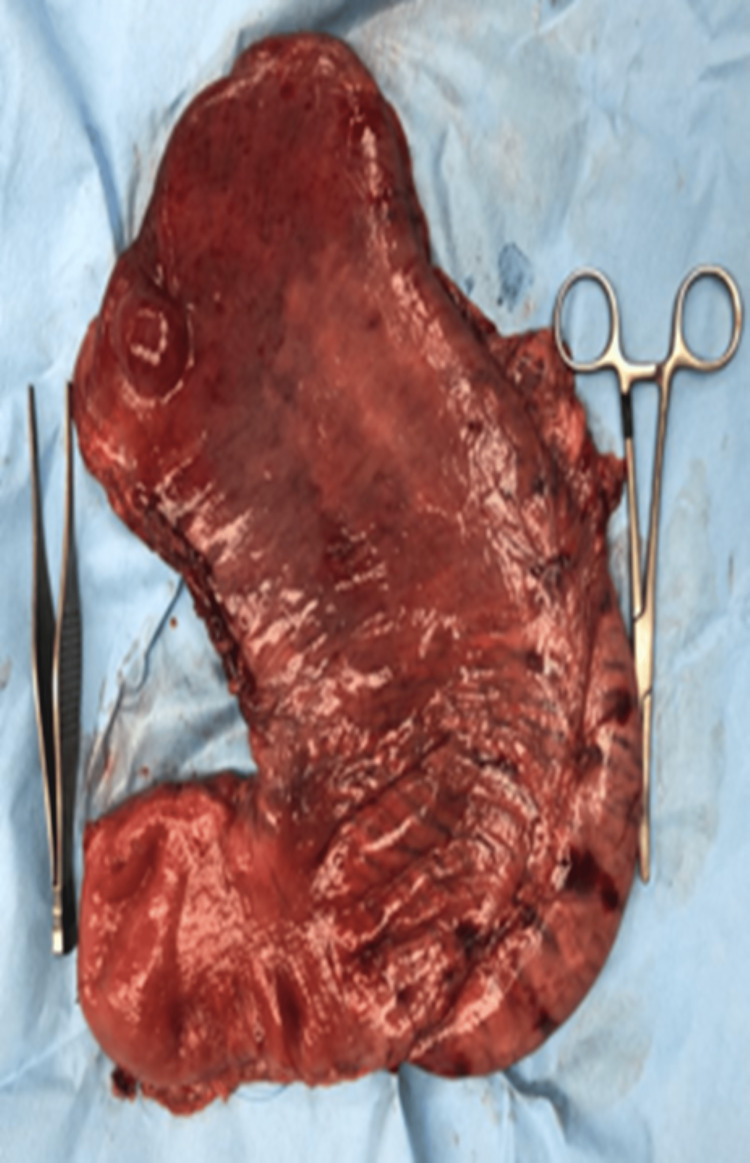
Image showing the total gastrectomy specimen

The gastric mucosa was hemorrhagic (totaling almost 1300ml blood loss) and the cardia, fundus, and body of the stomach were noted to be gangrenous. The antrum was noted to lie superior to the pylorus and appeared to be viable. The decision for a damage control total gastrectomy was made, and the procedure was performed for hemostatic and contamination control (Figure 5).

The distal esophagus and duodenal stump were stapled off with linear staplers, and the abdomen was left open with a Bogota bag applied.

The patient received 2 units of packed red blood cells and 4 units of fresh frozen plasma intraoperatively. However, he developed signs of disseminated intravascular coagulation immediately after surgery and subsequently died in the intensive care unit within 24 hours of the surgery.

## Discussion

Duplay first described the entity of acute gastric dilatation in 1833 [1] and later, in 1990, Casper et al documented its rarity: four cases in twenty-three thousand post-mortem specimens [2-4]. Acute gastric dilatation is characteristically described as the finding of a massively dilated stomach on radiological imaging. It is often associated with a high mortality (73-100%), particularly because this diagnosis may be easily overlooked [2, 4].

No cause has been identified in the pathophysiology of acute gastric dilatation, but several conditions have been associated with the development of this problem (Table 1) [2,4,5].

**Table 1:** Causes of acute gastric dilation

MECHANICAL	NON-MECHANICAL
Gastric volvulus	Invasive aspergillosis
Gastric outlet obstruction	Critical celiac trunk stenosis
Superior mesenteric artery syndrome	Psychogenic eating disorders (Atonic theory described by Brinton in 1859)
Post-surgical complications	Trauma: ingestion of corrosive substances
Intrathoracic herniation	Diabetic gastroparesis

The ischemia associated with acute gastric dilation is thought to be due to venous congestion and insufficiency. Even with a rich collateral blood supply, this may occur when intra-gastric pressures exceed gastric venous pressure (greater than 20-30cm H_2_O) or when the stomach is distended with three or more liters of contents [6].

First described in the 1990s by Kron et al, the World Society of Abdominal Compartment Syndrome later defined abdominal compartment syndrome as a sustained intra-abdominal pressure (IAP) over 20 mmHg (with or without an abdominal perfusion pressure < 60 mmHg) associated with new organ dysfunction or failure [7]. In our case, intra-abdominal pressure measurement prior to surgery was not possible as the patient was taken to the operating theater promptly. However, there remained a high index of suspicion for this complication as the patient had acutely developed a tensely distended abdomen with oliguria progressing to anuria and respiratory failure.

As postulated by Abdu et al, the catastrophic complication of rupture is caused by mucosal necrosis, followed by full-thickness ischemia of the gastric wall and perforation. Perforation occurs when intragastric pressures exceed 120mmHg or four liters of intragastric content [8]. The published literature suggests that this is located most often along the greater curvature and at the gastric fundus, with a mortality rate of up to 100% [1]

Characteristic clinical features include nausea and vomiting (which provides minimal relief), poorly localized abdominal pain, and distention [3]. In some cases, patients are reported to have an inability to vomit, which is not clearly understood. This may be due to occlusion of the gastroesophageal junction when the fundus distends, producing a one-way valve. Also, acute gastric volvulus is characteristically known to present with retching and an inability to vomit, and patients develop abdominal distension [1]. A sudden increase in intra-abdominal pressure leads to the development of compartment syndrome, which threatens the viability of organs [6].

Early radiological imaging, particularly abdominal X-ray, demonstrates a massively distended stomach with a fluid level, as illustrated in the case presented here. An X-ray may also indicate a pneumoperitoneum when perforation occurs. However, when rapidly available, computed tomography (CT) can also help identify the pathological cause [4]. In this case, the patient was not stable enough to allow the time to obtain a CT scan. The clinical information obtained was deemed sufficient to make a diagnosis and decide for surgical intervention.

Recognition of acute gastric dilatation and immediate decompression via nasogastric tube is the mainstay of treatment. Even after decompression, however, delayed perforation or bleeding is possible and this should be considered in planning treatment for these patients [5]. In our case, due to the presence of semisolid material in the stomach, a large nasogastric tube (18 Fr) decompression was inefficient; therefore, an immediate surgical intervention was mandatory [5]. Where patients are hemodynamically stable without signs of perforation, endoscopy is described as a useful adjunct in the evaluation of the stomach before definitive management. In experienced hands, upper endoscopy may also help untwist the stomach in acute volvulus to prevent the progression of gastric ischemia and spare the need for surgery [4].

When it comes to the ideal surgical approach, the underlying principle of resection of any gangrenous part of the stomach must be followed. Total gastrectomy with esophagojejunostomy and feeding jejunostomy is considered the procedure of choice, but it requires a considerable amount of operating time and a hemodynamically stable patient [1,2]. Other surgical procedures described in the literature include laparotomy for surgical decompression with drainage in cases of peritonitis and partial gastrectomy when the stomach is salvageable [2,9].

In our case, the patient was unstable, requiring high inotropic and ventilatory support settings. Due to the large lesser curvature perforation, persistent bleeding from the gastric mucosa, and full-thickness ischemia, a total gastrectomy without anastomosis appeared to be the only option for a damage control approach to treatment. To the best of our knowledge, this report appears to be the first documentation of total gastrectomy without anastomosis in a severely unstable patient with acute gastric dilatation. Few published case reports describe the successful completion of anastomoses in unstable patients with gastric dilatation and perforation [4].

According to Jung et al, despite a curtailed operative time with a damage control approach, acute gastric dilatation is still associated with high surgical mortality rates of 50-65% and up to 100% when not treated appropriately [4].

## Conclusions

Acute gastric dilation is a potentially lethal condition with a myriad of etiologies. While uncommon, it is necessary for the attending clinician to be able to recognize the features of acute gastric dilation and to initiate early and rapid resuscitation, which includes intravenous fluids and nasogastric decompression. In many cases, non-operative management is successful, but emergent surgery is undertaken when non-operative management fails or complications like necrosis and perforation occur. The documentation of this case report highlights the importance of a rapid diagnosis of acute gastric dilatation and the need for definitive management.
